# DNA Catalysis: Design, Function, and Optimization

**DOI:** 10.3390/molecules29215011

**Published:** 2024-10-23

**Authors:** Rebecca L. Stratton, Bishal Pokhrel, Bryce Smith, Adeola Adeyemi, Ananta Dhakal, Hao Shen

**Affiliations:** 1 Department of Chemistry and Biochemistry, Kent State University, Kent, OH 44242, USA; rstratt6@kent.edu (R.L.S.); bpokhrel@kent.edu (B.P.); bsmit219@kent.edu (B.S.); aadeyem3@kent.edu (A.A.);; 2 Advanced Materials and Liquid Crystal Institute, Kent State University, Kent, OH 44242, USA

**Keywords:** DNA catalysis, DNAzyme, DNA-nanoparticle hybrid, design, function, optimization

## Abstract

Catalytic DNA has gained significant attention in recent decades as a highly efficient and tunable catalyst, thanks to its flexible structures, exceptional specificity, and ease of optimization. Despite being composed of just four monomers, DNA’s complex conformational intricacies enable a wide range of nuanced functions, including scaffolding, electrocatalysis, enantioselectivity, and mechano-electro spin coupling. DNA catalysts, ranging from traditional DNAzymes to innovative DNAzyme hybrids, highlight the remarkable potential of DNA in catalysis. Recent advancements in spectroscopic techniques have deepened our mechanistic understanding of catalytic DNA, paving the way for rational structural optimization. This review will summarize the latest studies on the performance and optimization of traditional DNAzymes and provide an in-depth analysis of DNAzyme hybrid catalysts and their unique and promising properties.

## 1. Introduction

Nucleic acid catalysts have long intrigued scientists, bridging the fields of kinetics and supramolecular systems [1,2]. While protein enzymes were once considered the primary biological catalysts, Woese et al. proposed the idea of catalytically active RNA in the late 1960s [3]. This theory led to the discovery of ribozymes [4], with the key studies conducted by Thomas R. Cech and Sidney Altman, who demonstrated that RNA sequences could catalyze reactions without proteins [5]. These ribozymes are also referred to as “RNAzymes” to highlight the catalytic role of RNA. These early RNAzymes, capable of splicing and modifying phosphodiester bonds, paved the way for new research opportunities [6]. Over time, artificial selection and studies of native RNAs have expanded the number of known ribozymes [7,8,9,10,11,12]. Today, catalytic nucleic acids can be designed to selectively cleave RNA molecules, offering promise for gene expression control, infection prevention, and related therapies [13,14,15,16].

Since RNAzymes initially proved to be successful and modifiable nucleic acid catalysts, their existence sparked intriguing theories about the potential for DNA-based catalysts [7]. Unlike RNA, which typically exists as short, single-stranded segments with accessible active sites due to unpaired nucleotides folding into helical structures [17,18], DNA was initially considered less suitable for catalysis. This skepticism stemmed from DNA’s structural characteristics: the absence of the 2′-OH group, which limits the formation of certain catalytically active conformations, and its common double-stranded form with fewer unpaired nucleotides [19]. Moreover, the lack of naturally occurring DNAzymes presents further challenges in designing DNA catalysts. However, these very structural differences have since proven advantageous, enabling DNAzymes to compete impressively with their RNAzyme counterparts in versatility and effectiveness [20].

Following the initial expansion of RNAzyme research, G. F. Joyce and R. R. Breaker leveraged the existing theoretical framework of ribozyme behavior to synthetically create the first known RNA-cleaving DNAzyme [21]. This DNAzyme, now known as the 10–23 DNAzyme, has since been applied in various contexts [22]. As anticipated, the list of known DNAzymes has grown substantially, with the universal database “DNAmoreDB” now containing hundreds of entries [23]. Currently, DNAzymes go beyond cleaving oligonucleotides at specific sites [24,25,26]. They can facilitate reactions such as ligation [27,28,29], phosphorylation [30,31], deglycosylation [32], and acylation [33]. They serve as effective mimics of many natural enzymes, including esterases [34,35], laccases [36], photolyases [37,38,39], phosphoserine lyases [40], phosphatases [41], tyrosine kinases [42], chelatases [43,44,45,46], and particularly peroxidases [47,48]. In addition, DNAzymes have shown promise in modifying peptide chains through side-chain modifications, linkages, and elimination reactions [49,50,51], offering significant potential for targeted protein editing.

Beyond these functions, DNAzymes have been shown to catalyze a variety of chemical reactions, including the cleavage of anilides and aromatic amides [35], thymine dimer photoreversion [52], acylation of amines and lysines [53], oxidation of L-tyrosine and amyloid β [54], reductive amination [55], enantioselective Diels–Alder reactions [56], Friedel–Crafts alkylation [57], Michael addition [58], metal oxide degradation [59], aldol reactions [60], and others [61]. Even more intriguing is the emerging practice of combining DNAzymes with other types of materials, successfully integrating both organic and inorganic components [62].

The rapid expansion of available DNAzymes can be largely attributed to the development of the in vitro selection method (also known as directed evolution or SELEX) method in the 1990s [63,64,65,66]. This process typically involves multiple rounds of selection followed by amplification, ensuring the precise identification of the most effective DNAzymes [67]. According to DNAzyme expert Silverman, the success of in vitro selection features a key advantage of DNA catalysts over proteins: their sizes [68]. DNA consists of just four chemically similar bases—guanine (G), thymine (T), adenine (A), and cytosine (C)—while proteins are composed of a much larger and more diverse array of amino acids. This vast diversity in proteins, while potentially offering numerous catalytic functions, makes them practically impossible to evaluate and select through random methods like in vitro selection [69,70,71].

Despite the utility of in vitro selection, it has limitations, particularly in the development of large, complex DNAzymes. Some reactions may be too complicated for DNAzymes selected through in vitro techniques [61,66,72,73]. Nevertheless, in vitro selection remains a valuable method for discovering new DNAzymes, and more targeted approaches, such as chemical evolution, have emerged to refine and improve existing DNAzymes rather than restarting the selection process [24,74,75]. In recent years, research on catalytically active DNA has become more advanced and specialized, making the design, function, and optimization of DNAzymes essential for further development. One of the main challenges in improving DNAzymes is the limited understanding of their sequence-specific activity, conformational dynamics, and behavior within larger molecular complexes. Recent breakthroughs have begun to address these gaps, but few review articles focus on comparing the attributes of DNAzymes, particularly in the context of supramolecular complexes.

The purpose of this review is to systematically organize catalytic DNA research, starting with a focus on DNA-based catalysts that function primarily due to their inherent DNA structure, without the influence of additional materials. This subgroup allows for the exploration of sequential, environmental, conformational, and mechanical modifications without the confounding effects of hybrid structures. Subsequent sections will delve into the design, function, and optimization of DNAzyme hybrid structures. First, we will explore the integration of DNA with inorganic materials, creating DNA–nanocore hybrids. Later, we will examine hybrids that incorporate soft matter or biopolymers like DNA, RNA, and proteins.

Notably, metal nanoparticles such as gold (Au), platinum (Pt), and silver (Ag); metal oxides like iron oxide (Fe_2_O_3_/Fe_3_O_4_), manganese oxide (Mn_2_O_3_), and titanium dioxide (TiO_2_); and carbon-based materials like graphene oxides, carbon nanotubes, and fullerenes have been combined with DNA to generate hybrid catalysts [76,77,78,79,80,81,82,83,84,85,86,87]. These inorganic nanoparticles, known as nanozymes, often exhibit catalytic activities on their own [88,89,90,91]. When assembled with DNA, these nanozymes can achieve enhanced catalytic performance through complex and sometimes unexpected interactions. This synergy not only complements the existing strengths of DNA catalysts but also mitigates undesirable behaviors, highlighting the importance and accessibility of modern DNAzyme applications.

## 2. DNA-Dominant Catalysts

**Figure 1:**
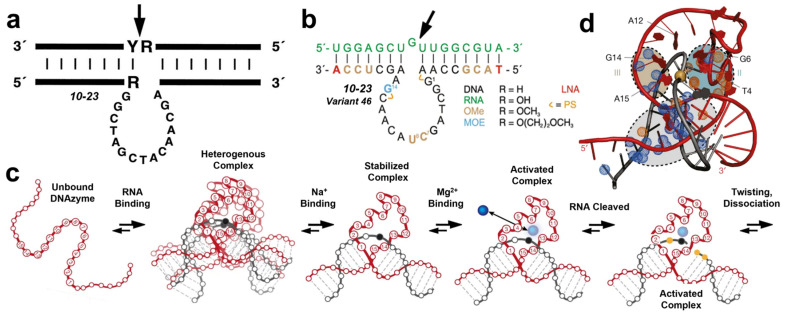
(**a**) The initial structure of an RNA-cleaving DNAzyme [6]. (**b**) A more modern render of the specific augments applied by Nguyen et al. in order to optimize 10–23 DNAzyme activity [98]. R-groups describe the chemical additions to the sugar backbone that create each respective DNA/RNA/OMe/MOE monomer. (**c**) The innovative spatially and temporally resolved mechanism of 10–23 DNAzyme activity proposed by Borggräfe et al. [99]. (**d**) A schematic illustration published by Borggräfe et al. Roman numerals show Sites I, II, and III [99]. Adapted from Refs. [6,98,99] with permission.

The complete mechanism of activity for the 10–23 DNAzyme—from its unbound aptamer state to the pre-dissociation activated complex—is now available as a theoretical framework for designing, understanding, and optimizing DNAzymes. Like other catalysts, DNAzymes must perform multiple tasks during each catalytic cycle: substrate binding, catalysis, and product release [92]. Different regions of the DNAzyme structure are typically responsible for each of these steps [93,94,95]. Most DNAzymes exhibit two key features: binding arms and a catalytic “motif” or active sequence [1,96]. Figure 1a illustrates the two-dimensional structure of a basic RNA-cleaving DNAzyme, with the cleavage site indicated by an arrow. The binding arms are represented by the 5′ to 3′ strand, while the loop of bases below the structure shows the active catalytic sequence [21]. Binding arms are particularly adept at interacting with oligonucleotide substrates, primarily serving to “capture” and orient the substrate in the ideal position for catalysis [1]. The catalytic motifs, on the other hand, are often used as broader categories for comparing the mechanisms of different DNAzymes [97]. The binding arms surrounding each motif can be strategically altered to modify the DNAzyme’s substrate specificity [32].

Several variants of the RNA-cleaving 10–23 DNAzyme have been developed to improve catalytic performance. Figure 1b illustrates the two-dimensional structure of the most effective variant, Dz46. In this study, Nguyen et al. incorporated 2′-OMe, 2′-MOE, LNA, and phosphorothioate modifications into Dz46’s catalytic core [98]. These modifications enabled Dz46 to perform over sixty catalytic turnovers within 30 min in conditions closely resembling physiological environments. The authors further demonstrated that Dz46 is highly effective as an allele-specific gene-silencing agent, even against targets previously considered undruggable.

The 10–23 DNAzyme exemplifies the use of SELEX or chemical evolution in the informed optimization of catalysts. Figure 1c depicts the stepwise model provided by Borggräfe et al., with the DNA catalyst and RNA substrate shown in red and black, respectively [99]. To validate the accuracy and significance of this structural model, Borggräfe et al. employed a “rationally selected” single-atom replacement mutation, which led to a six-fold increase in catalytic activity. This simple yet strategic modification significantly enhanced the DNAzyme’s performance, highlighting the potential of well-informed design adjustments.

Many DNAzyme species rely heavily on cofactors to adopt proper global folding patterns necessary for their active conformations [100,101]. Common cofactors include metals such as lead, magnesium, sodium, potassium, and lithium, or combinations of these [22]. Modifications to a DNAzyme can also affect its cofactor preferences, potentially shifting from a dependency on two metals to a reliance on just one. Larger molecules like hemin [59], serotonin [52], histidine [36], and even lanthanides [102] have also been found to enhance catalytic activity.

Despite their importance, cofactors are challenging to study due to their weak and dynamic interactions with DNA catalysts. However, recent advancements in DNAzyme imaging, such as those by Borggräfe et al., have shed light on these interactions [99]. Specifically, the roles and locations of cofactors within the 10–23 DNAzyme system have been identified with a fair degree of accuracy. NMR titration studies have revealed a structure, shown in Figure 1d, that highlights three metal-binding sites within the DNAzyme. The ion at Site I reduces repulsion between the phosphate backbones in the binding arm region. The ion at Site II triggers “conformational activation” on the 5′ side of the catalytic loop, while the ion at Site III aligns the 3′ side of the catalytic loop with the scissile bond, promoting cleavage.

Furthermore, various environmental factors, such as temperature, solvent, and pH, can be adjusted to influence DNAzyme activity [103,104,105,106,107,108]. For instance, Li et al. demonstrated that adding butanol enhances NaA43T DNAzyme activity [103]. Other studies have shown that DNAzymes can function effectively across a range of temperatures, from high to room temperature [104,105,106]. Additionally, pH plays a crucial role in regulating DNAzyme function [107], with some DNAzymes operating in highly acidic conditions even in the absence of cofactors [108]. These environmental adjustments work because the correct positioning of DNA’s active sites is essential for catalytic activation. As illustrated in Figure 1c,d, the three-dimensional structure of the catalytic motif must be properly oriented for a DNAzyme to function.

**Figure 2:**
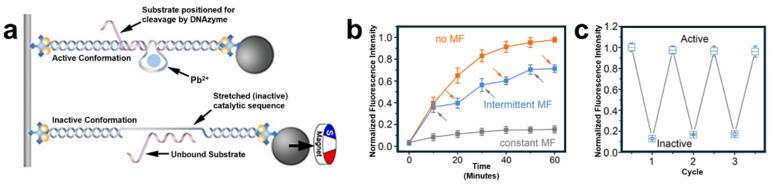
(**a**) Magnetic bead set-up used by Li et al. to stretch the active site of a DNAzyme into an inactive conformation [109]. (**b**) A graph representing the effect of magnetic field (MF) application on the activity of catalysts. Products were modified with a fluorescent probe to track their formation via intensity. (**c**) Alternate representation of activity and MF relationship per “cycle” MF of activation. Adapted from Ref. [109] with permission.

An intriguing 2022 study by Li et al. used a magnetic bead to cyclically toggle a DNAzyme’s activity manually and repeatedly [109]. In the experimental design shown in Figure 2a, a magnetic bead was attached to the binding region of either a lead- or magnesium-dependent DNAzyme. By applying an upward magnetic field, the DNAzyme was activated or deactivated continuously or intermittently. The resulting fluorophore-labeled product detection provided the data shown in Figure 2b,c. This study highlights that distortion of a DNAzyme’s active sequence can hinder or completely block catalytic reactions. When the key bases of a DNAzyme are stretched into an inaccessible position, substrate molecules are either unable to bind or unable to react as they would with an undistorted DNA sequence.

Apart from external modifications, internal changes to DNA structures can be achieved through chemical evolution. These changes may include sequence mutations or chemical modifications, such as introducing functional groups to the bases [61,66,92,110,111,112,113,114,115,116,117,118,119,120,121,122]. The synthesis of modified nucleoside triphosphates (dNTPs) has gained considerable interest due to its numerous advantages. Hollenstein described this approach as “elegant” for enhancing DNA-based enzyme-mimicking catalysts [117]. Modifications to dNTPs can involve alterations to the sugar, base, or phosphate groups of nucleotides. These enhancements are often designed to improve the properties of DNA catalysts or to introduce new functionalities, such as incorporating non-standard bases or sugar analogs [123]. For example, phosphorothioate bonds, which replace a non-bridging oxygen atom in the phosphate backbone with sulfur, have been used to increase stability.

**Figure 3:**
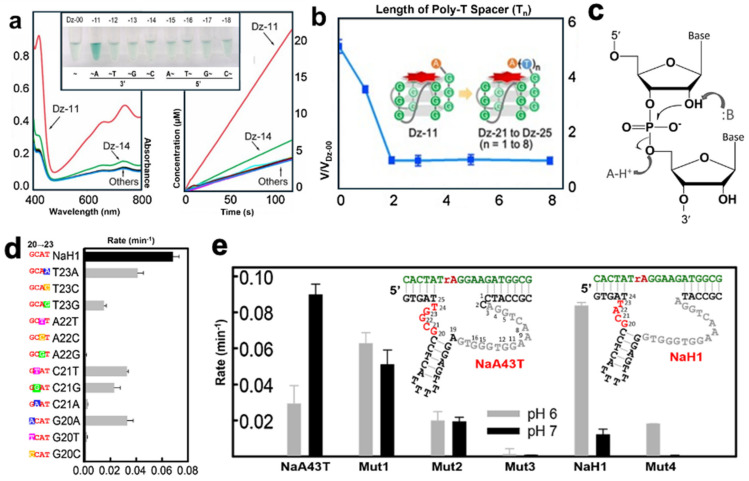
(**a**) Modifications to the 3′ and 5′ ends of Dz-00 and the activity variation for the mutated species (Dz-11 to Dz-18). (**b**) Decrease in activity due to increase in spacer length. (**c**) A mechanism for DNAzyme-catalyzed cleavage of oligonucleotide monomers. (**d**) Single point mutations to the four main catalytic bases of NaH1 (left) and their respective rates. (**e**) A comparison of NaA43T to NaH1 at different pH values, including other mutations showing less significant results. Adapted from Refs. [113,124] with permission.

G-quadruplex DNAzymes primarily function as peroxidase mimics and exemplify sequence-dependent catalysis [124]. In a 2016 study by Li et al., the guanine-rich DNAzyme Dz-00 was modified to assess the impact of adjacent adenine on its catalytic activity. Figure 3a shows that adding adenine to the 3′ end, forming Dz-11, significantly enhanced activity, while Dz-14, with an adjacent cytosine, showed a slight increase, likely due to its chemical similarity to adenine. Li noted that adenine’s effect is similar to distal histidine in peroxidase enzymes (Figure 3b). When a spacer chain was introduced between adenine and Dz-11’s active site, activity decreased dramatically, indicating that even small sequence changes can greatly improve turnover rates.

Despite the lack of a fully resolved 3D structure, sequence modifications have revealed the key roles of specific bases in DNAzyme function. Li et al.’s work suggests the unprotonated form of adenine’s N1 likely optimizes Dz-00’s performance. Their modifications imply that Dz-00 and Dz-11 operate via a general acid–base mechanism, a theory supported by other studies [125]. Beyond refining known mechanisms, sequence modifications also raise the potential for altering a DNAzyme’s function and its activity conditions [126].

This versatility highlights the complexity of DNAzymes. In the realm of RNA-cleaving DNAzymes, the 8–17 motif has drawn attention alongside the well-known 10–23 DNAzyme [127]. The NaA43 DNAzyme, introduced in 2015 by Lu et al., likely cleaves substrates via a general base mechanism (Figure 3c). Remarkably, Ma et al. discovered that a single-point mutation in NaA43 produced NaH1, a species that operates through a general acid mechanism under different pH conditions [113]. Ma et al. conducted point mutations on a four-base segment of NaH1 (Figure 3d), revealing the importance of specific bases in its catalytic activity. They also demonstrated NaH1’s ability to match NaA43’s cleavage rate but at a much higher pH, with only one base change near the catalytic sequence (Figure 3e).

**Figure 4:**
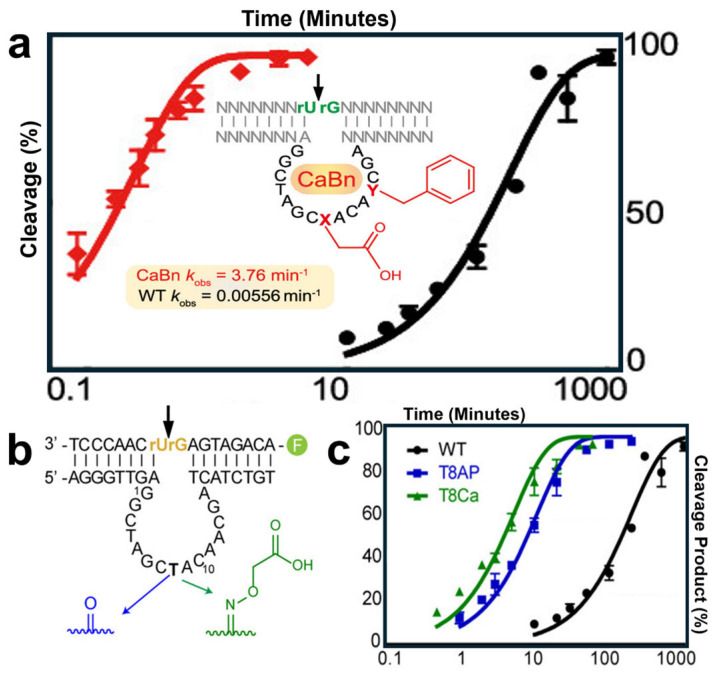
(**a**) Red line shows the cleavage percentage of modified CaBn species compared to the wild type DNAzyme represented in black. Inset: specific locations and structures of modifications, carboxyl and benzyl groups. (**b**) Selection of two possible installations at position T8. (**c**) The comparison of each modification’s activity, with carboxyl (green) showing the best cleavage product to time relationship. Adapted from Ref. [128] with permission.

The introduction of functional groups to the active sites or adjacent bases of a DNAzyme can significantly influence its catalytic behavior. In a recent study published by Zhang et al., researchers reported an impressive 700-fold increase in the efficiency of the 10–23 DNAzyme after adding two small functional groups to its catalytic motif, generating the CaBn species [128]. Carboxyl and benzyl groups were attached to positions 8 and 12 (Figure 4a), selected based on insights from previous structural studies of the 10–23 DNAzyme. Figure 4b illustrates the initial modifications made solely at position 8, chosen due to the known tolerance of T8 to mutations [129,130]. Among the modifications, the carboxyl group (green line in Figure 4c) had the most substantial impact on DNAzyme activity. Interestingly, while the functional groups shown in Figure 4b exhibited significant individual effects, Zhang’s study demonstrated that the combined installation of these groups resulted in a near-exponential enhancement of catalytic performance.

Borggräfe et al. provided further insights suggesting that the CaBn species enhances activity by increasing the number of magnesium ions available at the DNAzyme’s “Site II”, thereby promoting more active conformations within the ensemble. Notably, this enhanced ability to capture and interact with magnesium ions reduces the DNAzyme’s reliance on high cofactor concentrations. Similar findings related to cofactor density have been reported in other studies [131]. Zhang’s chemoenzymatic modification study parallels an earlier optimization study by Nguyen et al., though Zhang’s work achieved a greater activity boost through a simpler modification process. These findings reinforce the synergistic nature of DNAzyme optimization and highlight the promising potential of this field.

Given the diverse reactions facilitated by known DNAzymes and the flexibility of single-stranded DNA aptamers, it may also be possible to expand DNAzyme applications by substituting aptamers more suited to specific conditions. For instance, one challenge faced by many G-quadruplex catalysts is their inability to selectively recognize porphyrins, along with their tendency to bind undesirable planar molecules [132,133,134]. Such behavior limits their usefulness in environments where non-reactive molecules could monopolize or deactivate binding sites [135].

**Figure 5:**
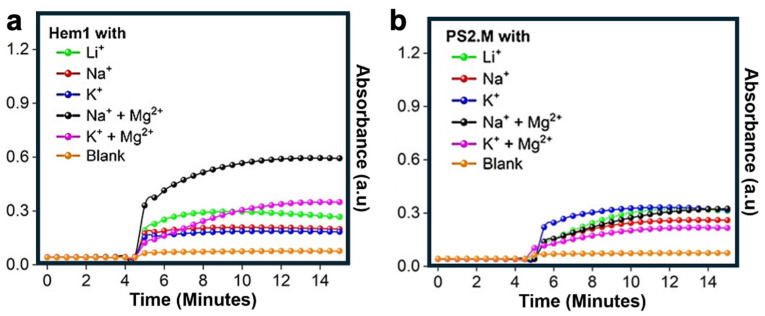
(**a**) The absorbance and time relationship for the Hem1 aptamer complex with six different cofactor environments. The sodium and magnesium pairing vastly increases activity to roughly double PS2.M’s highest activity with any cofactors. (**b**) The absorbance and time relationship for PS2.M aptamer complex with six different cofactor environments. Potassium shows the most promising results but remains at roughly 0.3 a.u. Adapted from Ref. [135] with permission.

In a 2024 study, Gu et al. provided an excellent example of aptamer substitution by comparing two DNA catalysts: Hem1 and PS2.M. Hem1, which was developed using SELEX to specifically avoid forming a G-quadruplex structure, was confirmed by multiple spectroscopic techniques to retain this property. As illustrated in Figure 5a, its activity was evaluated in the presence of various cofactors. In contrast, PS2.M, a classical G-quadruplex species, exhibits the common limitations of G-quadruplex catalysts, such as reduced activity and distinct cofactor preferences, as shown in Figure 5b. By substituting the traditional G-quadruplex aptamer PS2.M with the rationally designed Hem1, Gu et al. successfully overcame the structural challenges of PS2.M and achieved a notable increase in catalytic activity.

The 10–23 DNAzyme has emerged as a leading candidate in ushering the modern era of rational optimization. Other DNA catalysts are following closely behind, with studies focusing on the structural optimization of the 8–17 DNAzyme, peroxidase-mimicking DNAzymes, and various others, yielding equally impressive results over the past decade [136,137,138,139]. With the availability of comprehensive three-dimensional structures for more catalytic motifs, progress in this field is expected to accelerate significantly.

Strategies such as in vitro selection, chemical evolution technologies, environmental adjustments, sequence modifications, functional group installations, nucleotide alterations, mechanical manipulations, and aptamer substitutions have all demonstrated promising results. However, these optimization techniques are limited by the inherent constraints of DNA-dominant catalysts. To address this, researchers have explored hybridizing DNA with other materials, aiming for more substantial modifications and enhanced optimization.

## 3. DNA–Nanoparticle Hybrid

Nanozymes are nanoparticles with enzyme-like properties, capable of converting various substrates into products. When combined with DNA, they form a new class of catalysts, DNAzyme/nanozyme hybrids, that provide a high surface area, multiple catalytic sites, enhanced reaction specificity, and biocompatibility [91,140,141,142,143]. These hybrids often exhibit modified behavior compared to the parent nanozymes or DNAzymes, as the nanoparticle and DNA components can serve distinct functions during catalysis. This allows researchers to independently optimize both components for enhanced performance. Sometimes, a synergistic effect occurs at the DNA–nanoparticle interfaces. DNA–nanoparticle hybrids can be synthesized through techniques such as thiol-gold linkages [144], click chemistry [145], and noncovalent binding to the nitrogenous bases of DNA [62,146].

**Figure 6:**
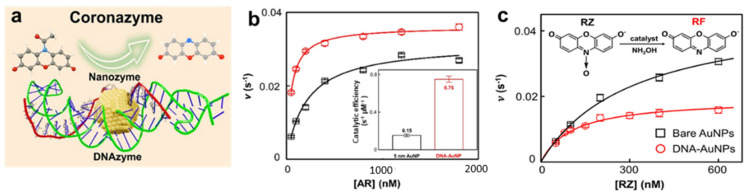
(**a**) Schematic diagram of the into coronazyme structure and catalysis. (**b**) Activity comparison between bare 5 nm AuNPs and coronazymes for the oxidation of amplex red. Inset: Catalytic efficiency of 5 nm AuNP compared to AuNP@DNA coronazyme. (**c**) Activity comparison between bare 5 nm AuNPs and coronazymes for the reduction in resazurin. The coronazyme suppresses the resazurin activity by blocking its access to AuNP. Adapted from Ref. [62] with permission.

A notable example of this approach was demonstrated by Shen and Mao, who linked DNA hairpins to gold nanospheres (AuNPs, Figure 6a) [62]. The DNA formed a corona-like structure around the AuNP surface, leading them to name this catalyst the “coronazyme”. Both the original AuNPs and the resulting coronazyme function as peroxidase mimics, catalyzing the oxidation of fluorogenic amplex red in the presence of hydrogen peroxide. However, the DNA-functionalized coronazyme exhibited a five-fold increase in catalytic efficiency compared to bare AuNPs (Figure 6b). Kinetic analyses and density functional theory (DFT) calculations showed that this improved performance stems from the strong interaction between the DNA and the substrate, enabling long-range catalysis exclusively within the DNA corona. This interaction transforms individual DNA bases into reactive sites, enhancing the coronazyme’s substrate selectivity. For instance, while resazurin is structurally similar to amplex red, the coronazyme’s activity in converting resazurin is significantly lower (Figure 6c), as the binding between resazurin and the coronazyme is weaker. This selective binding behavior allows the coronazyme to mimic enzymes by modulating its binding strength toward different substrates, depending on the surrounding DNA structure.

**Figure 7:**
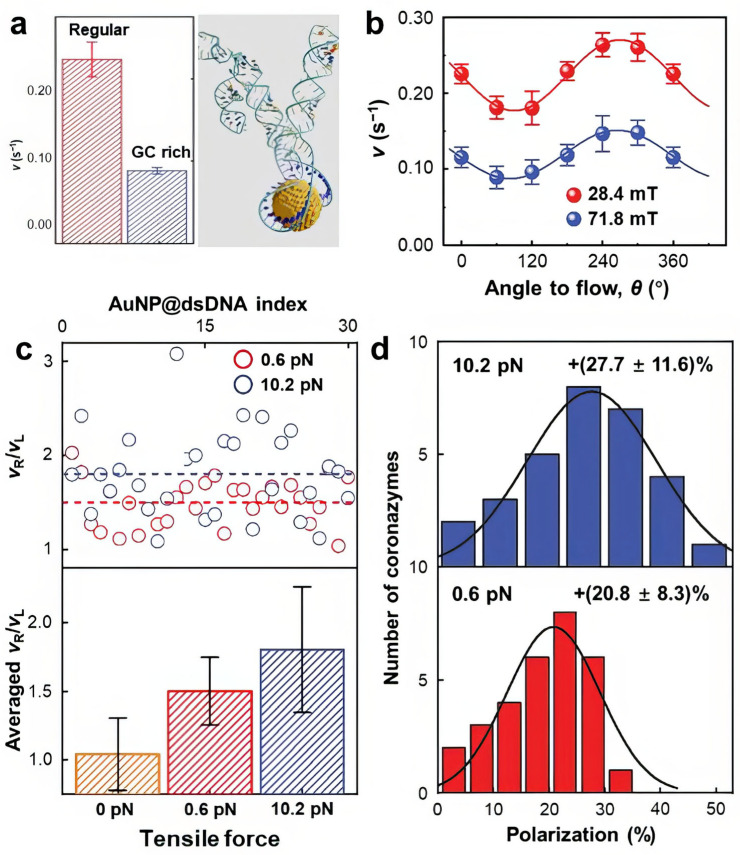
(**a**) Comparison of the coronazyme activity between the randomized based pair sequence and a GC-enriched sequence. (**b**) Magnetic field orientation-dependent coronazyme reactivity. (**c**) Top: activity ratio for individual coronazymes under RHCP and LHCP radiation. Bottom: averaged activity ratio (RHCP/LHCP) under various tensile force conditions. (**d**) Calculated activity polarization based on the RHCP/LHCP ratio shown in (**c**). Top: activity polarization under 10.2 pN. Bottom: activity ratio under 0.6 pN. Adapted from Ref. [147] with permission.

The authors further discovered that the catalytic performance of coronazymes is highly dependent on the DNA sequence [147]. A regular DNA hairpin with randomized bases showed higher activity compared to a GC-enriched hairpin (Figure 7a), as the latter tends to immobilize charges within it. This finding suggests that DNA acts as a charge conduit, and its charge-conducting capability directly influences the catalytic activity during redox reactions. Similar to DNA–hemin systems, internal charge transduction occurs within the coronazyme, beginning with charge injection from the AuNP to the DNA hairpin. Since the reaction substrates bind exclusively to the DNA bases, charges must transfer through the DNA strand to reach the bound substrate. Previous studies have shown that DNA can transfer charges over distances of tens of nanometers, but this charge conduction is sequence-dependent. Therefore, the activity of the coronazyme is closely linked to the DNA sequence.

Additionally, because DNA is intrinsically chiral, electron spin is modulated as charges pass through the DNA strand [148,149,150]. This recently discovered phenomenon is known as chiral-induced spin selectivity (CISS) [151,152]. The CISS effect suggests that DNA strands can act as both electron spin inducers and filters when attached to nanoparticles. The chiral structure of DNA, along with its preference for specific electron spins, can be leveraged to enhance the overall catalytic performance of DNA-based catalysts. This interpretation was supported by Shen and Mao’s findings, where they observed that DNA-wrapped coronazymes responded to external magnetic fields, as electron spins were aligned to the field direction at the Au-DNA interface (Figure 7b) [147]. Moreover, circularly polarized light (CPL) can generate electron spin polarization at this interface. Notably, when exposed to right-hand circularly polarized light (RHCP), which matches the chirality of DNA, the coronazyme exhibited consistently higher activity compared to left-hand circularly polarized light (LHCP) (Figure 7c). Beyond utilizing magnetic fields and CPLs to modulate coronazyme performance, the researchers also applied mechanical forces to stretch the DNA hairpin during catalysis. The coronazyme’s activity responded to these force stimuli, as the change in DNA conformation altered its chirality and, consequently, its charge-conduction capabilities (Figure 7d). These discoveries demonstrate that DNA-based hybrid catalysts can respond to various external stimuli, resulting in modulated catalytic performance.

**Figure 8:**
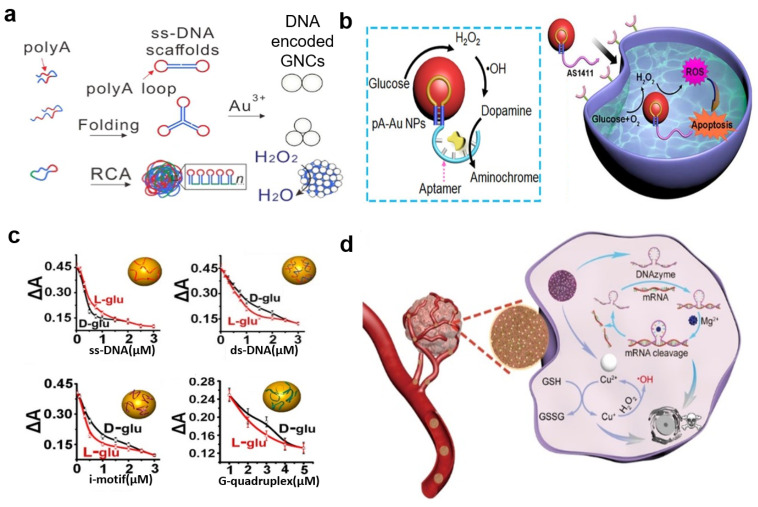
(**a**) Schematics for ss DNA scaffold programmed Gold Nanoparticle Clusters (GNCs) for their peroxidase activity. (**b**) Schematics of the DNA aptamer-modified Au nanoparticles for dual catalytic activity of H_2_O_2_ mediated dopamine oxidation and aerobic oxidation of glucose for chemodynamic cancer treatment. (**c**) DNA concentration-dependent chiral selective catalysis of the AuNPs with ssDNA (top left), dsDNA (top right), imotif (bottom left), and G-quadruplex (bottom right). (**d**) Schematics for ss DNA scaffolds programmed gold nanoparticle clusters (GNCs) for their peroxidase activity. Adapted from Refs. [153,154,155,157] with permission.

The peroxidase-like activity of DNA-AuNP hybrids was also explored by Hizir et al., who capped AuNPs with single-stranded DNA (ssDNA) [78]. They found that the ssDNA-AuNP system exhibited significantly enhanced TMB oxidation as the negatively charged phosphate backbone of the ssDNA facilitated the electrostatic attraction of the substrate. This highlights DNA’s ability to enhance catalysis by promoting substrate adsorption. Similarly, Chen et al. synthesized ssDNA-encoded gold nanoparticle clusters (GNCs) as programmable enzyme equivalents (PEEs) (Figure 8a) [153]. These ssDNA scaffolds assemble into folded nanostructures with polyadenine (polyA) loops and double-stranded stems acting as nucleation sites, leading to increased binding affinity for reaction substrates.

DNA’s intrinsic chirality has also been harnessed for substrate recognition. Recently, Ouyang et al. modified DNA into a dopamine-binding aptamer (DBA) and conjugated it to polyadenine-stabilized Au nanoparticles (pA-AuNPs) to create an aptananozyme (Figure 8b) [154]. This aptananozyme catalyzed H_2_O_2_-mediated dopamine oxidation to aminochrome through the aerobic oxidation of glucose. Compared to separate nanozyme/aptamer units, the aptananozyme showed a 10-fold increase in dopamine oxidation by H_2_O_2_ and a 13-fold increase in the presence of glucose. This remarkable enhancement was attributed to the concentration of dopamine at the catalytic interfaces, facilitated by chiral-selective aptamer–dopamine binding.

Similarly, Zhan et al. reported the use of DNA-capped AuNPs as chiral-selective nano catalysts for glucose oxidase-mimicking reactions [155]. Uncapped AuNPs showed no preference for glucose enantiomers, indicating that the chirality preference was introduced exclusively through the addition of DNA. The ssDNA-AuNPs displayed higher activity for L-glucose than D-glucose, due to the stronger interaction with L-glucose, which was likely driven by its stereo orientation preference. However, the dsDNA-AuNPs, i-motif-AuNPs, and G-quadruplex AuNPs showed more preference for the D-glucose (Figure 8c). Collectively, these studies underscore DNA’s potential in catalyst design, as it can effectively adsorb reaction substrates, enrich reactants at catalytic surfaces, and introduce chirality for selective reactions.

It is important to note that DNA–nanozyme hybrids are not limited to AuNPs; other metals, such as copper (Cu), iron (Fe), and zinc (Zn), are also commonly used. For example, Fu et al. reported platinum (Pt) nanozymes synthesized using G-C rich nucleation centers [80], while Wei et al. developed DNA-based FeCuAg nanoclusters [156]. Both of these DNA-nanoparticle hybrids demonstrated peroxidase-like activities. Additionally, Liu et al. designed Cu-DNAzyme nanohybrids for delivering DNAzymes and Cu^2+^ into cancer cells for combined catalytic therapy [157]. These nanohybrids exhibited enhanced cell membrane permeability and excellent loading capacity (Figure 8d). The catalytic 10–23 DNAzyme in the nanohybrids cleaved human vascular endothelial growth factor-2 (VEGFR2) mRNA, leading to gene silencing. Simultaneously, the glutathione-induced reduction of Cu^2+^ to Cu^+^ catalyzed the conversion of endogenous H_2_O_2_ into cytotoxic hydroxyl radicals, enabling dual-catalytic tumor therapy.

**Figure 9:**
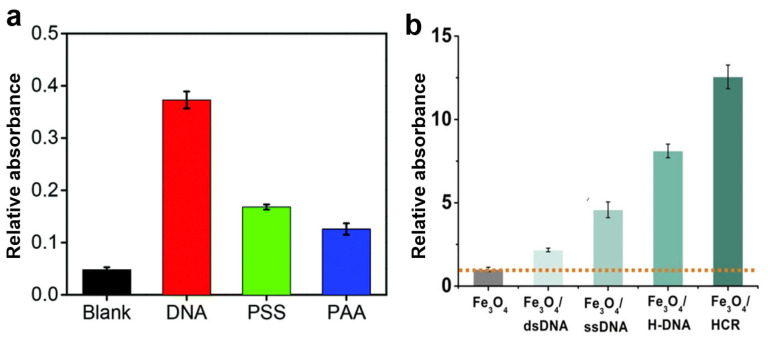
(**a**) Activity comparison of bare Fe_3_O_4_, with Fe_3_O_4_ modified with DNA, polystyrene sulfonate (PSS), and polyacrylic acid (PAA). (**b**) Relative catalytic rate for TMB oxidation for Fe_3_O_4_/dsDNA, Fe_3_O_4_/ssDNA, Fe_3_O_4_/H-DNA, and Fe_3_O_4_/HCR. Adapted from Refs. [77,82] with permission.

It is also common to hybridize DNA with metal oxides, such as Fe_3_O_4_ and TiO_2_ [77,158]. The rationale behind DNA–metal oxide hybrids is similar to that of DNA–metal catalysts: the presence of DNA enhances substrate interaction, thereby improving the overall catalytic activity. For example, Zhang demonstrated the use of DNA with iron cobalt oxide nanosheets (FeCo-ONs) as a peroxidase mimic [159]. In a study by Liu et al., DNA-capped Fe_3_O_4_ nanoparticles showed a roughly 10-fold increase in activity compared to bare nanoparticles (Figure 9a) [82]. The DNA ligands outperformed negatively charged polymers, such as polyacrylic acid (PAA) and polystyrene sulfonate (PSS), in nanoparticle modification, underscoring DNA’s superior substrate interactions. Beyond their negatively charged backbones, DNA molecules feature additional hydrogen bonding capabilities and π-π stacking interactions with substrates, significantly enhancing their binding properties. In a study conducted by Zhang and his colleagues, it was found that the peroxidase-like activity of ssDNA-Fe_3_O_4_ was doubled, dsDNA-Fe_3_O_4_ exhibited a 4.6-fold increase, hairpin DNA-NP demonstrated an 8-fold increase, and the hybridization chain reaction (HCR) H-DNA-Fe_3_O_4_ revealed a 13-fold enhancement compared to bare Fe_3_O_4_ (Figure 9b) [77]. The variations in DNA binding to the nanoparticles, attributed to different surface coverage, explain these differences in activity enhancement.

## 4. DNA–Carbon Hybrids

DNA can also be hybridized with various carbon materials to enhance their catalytic properties. Carbon-based materials, such as carbon dots [160] and graphene [161], have previously been explored for their high efficiency, large surface area, and versatility in different forms, making them ideal candidates for hybridization with DNA [162,163].

**Figure 10:**
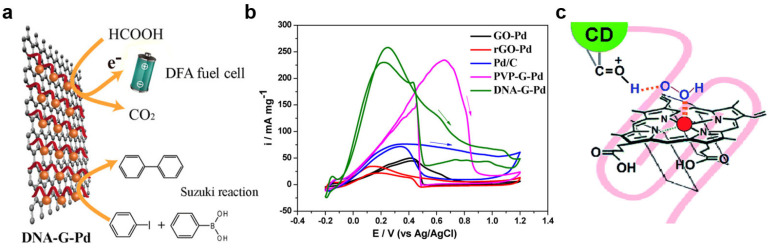
(**a**) Schematic of DNA-modified Graphene/Pd NPs (DNA-G-Pd) synthesis. (**b**) Cyclic voltammogram for oxidation of formic acid (**c**) Mechanism for CD–G quadruplex–hemin nanonetwork interaction for DNAzyme activity enhancement. Adapted from Refs. [164,165] with permission.

In a study by Qu et al., a DNA-modified graphene/Pd nanoparticle hybrid (DNA-G-Pd) was assembled for formic acid electro-oxidation and the Suzuki reaction (Figure 10a) [164]. These hybrids demonstrated higher catalytic activity, extended lifespan, and easy recyclability. Cyclic voltammetry (CV) analysis revealed that the mass-normalized peak current for DNA-G-Pd was 2.5 times higher than PVP-G-Pd and approximately 3.5 times better than Pd/C, highlighting the crucial role of DNA in electrochemical applications (Figure 10b). The DNA lattice not only distributed active sites evenly but also depleted oxygen in the solution, preventing Pd from forming passive PdO and supporting formic acid intermediates to promote catalysis. In another study by Das and his coworkers, a G-quadruplex/hemin network crosslinked by carbon quantum dots showed increased catalytic activity and enhanced stability (Figure 10c) [165]. The carbon dots (CDs) interacted non-covalently with the hemin/GQ network, facilitating electron transfer through keto carbonyl functional groups and creating a confined yet beneficial microenvironment for the ABTS oxidation reaction, leading to a faster catalytic rate.

Li et al. developed a nanocomposite composed of platinum, ssDNA, and reduced graphene oxide (ssDNA-RGO/cf-Pt), which exhibited 2.5 times greater catalytic activity than RGO-Pt and 3.8 times higher activity than regular Pt nanoparticles for methanol oxidation [166]. In methanol oxidation, CO adsorption typically poisons the catalytic efficiency, but the additional oxygen groups in the ssDNA and residual oxygen species in the RGO enhanced CO oxidation, providing resistance to CO poisoning. The anti-poisoning ratio was 1.75 times higher than that of RGO/Pt and Pt nanoparticles. These studies highlight the diverse role of DNA in generating superior catalysts and enhancing catalytic performance.

## 5. DNA–Soft Matter Catalysts

It is common practice to hybridize DNAzymes with natural biopolymers like peptides and synthetic polymers to create hybrid catalysts [167,168]. For instance, Ding and his colleagues synthesized a DNA/peptide nanoparticle that exhibited enhanced peroxidase-like activity through a synergistic mechanism [169]. The incorporation of histidine residues from the peptides facilitates hydrogen bonding, mimicking the role of distal arginine residues found in natural peroxidases. This interaction stabilizes the hemin aggregates on the guanine quartet of the DNA framework, enabling the hybrid to demonstrate superior peroxidase-like properties. The observed synergistic catalytic behavior stems from the complementary chemical and structural features of the peptides and DNA components.

**Figure 11:**
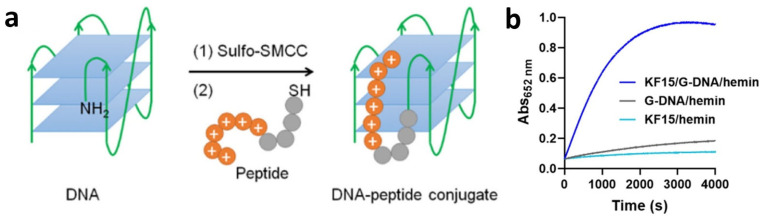
(**a**) Schematics of the DNA-peptide conjugate hybrid formation. (**b**) Activity comparison among peptide–DNA/hemin hybrid, G–DNA/hemin hybrid and peptide/hemin hybrid for TMB oxidation. Adapted from Refs. [170,171] with permission.

Similarly, Xiang and his team reported that cationic peptide conjugates covalently linked to DNAzymes resulted in a catalytically active DNA-peptide conjugate (Figure 11a) [170]. This conjugate exhibited increased peroxidase and oxidase activities, by up to fourfold and threefold, respectively. The enhancement was attributed to the electrostatic interaction between the peptides and DNA phosphates, as well as the π–π stacking between histidine and DNA nucleobases. These interactions stabilized the parallel DNA G-quadruplex structures and promoted hemin binding. Wang further demonstrated the engineering of a peroxidase-mimicking nanoparticle, utilizing hemin encapsulated between DNA G-quadruplexes and lysine-rich peptides (Figure 11b) [171]. This scaffolded architecture resulted in enhanced peroxidase-like activity due to the increased substrate binding capacity.

## 6. Conclusions

Significant progress has been made in DNA-based catalysts over the years. Initially a theoretical concept in the 1960s, catalytic nucleic acids have evolved into a versatile class of multifunctional catalysts. Recent advancements have generated various DNA-based catalysts, incorporating structural, conformational, and chemical modifications, aptamer engineering, and hybridization with metals, polymers, and biopolymers. These improvements have resulted in catalysts with higher stability, enhanced performance, better efficiency, chiral selectivity, and catalytic modulation via light, magnetic fields, or mechanical force. This progress offers enormous potential for DNA-based catalysts to address the limitations associated with natural enzymes. Further research into the dynamics and functional mechanisms of non-hybridized DNAzymes will help unlock the full potential of DNA-dominant catalysts and strengthen the foundation for optimizing DNA hybrid catalysts.

The selection of new DNAzymes via in vitro selection and chemical evolution is well-documented and has been enriched by advances in three-dimensional structure-resolution technologies. Understanding DNA’s activity across pH, temperature, solvent, cofactor, and substrate variations has been extensively reviewed. Mutation studies have identified key bases that must be conserved for catalytic activity. These insights, combined with spatially resolved, catalytically active DNAzyme structures, have revealed unexpected mechanisms. Simple functional modifications have improved DNAzyme activity up to 700-fold.

DNA’s biocompatibility makes it ideal for hybridization with other catalysts, combining catalytic traits to create superior hybrid catalysts. DNAzyme-hybrid catalysts, particularly those with metallic nanoparticles, represent a significant advancement due to their enhanced properties and versatility. Gold nanoparticles, for instance, conjugated with DNAzymes, can serve as sensitive biosensors or effective catalysts in redox reactions. These hybrids leverage the selective nature of DNAzymes and the catalytic and optical properties of nanoparticles. Incorporating other materials can further improve stability and catalytic activity, making DNAzyme hybrids suitable for challenging chemical environments. The precise control over nanoparticle size, shape, and composition offers another layer of optimization for DNA hybrid catalysts, enhancing performance in fields like environmental sensing, diagnostics, and drug delivery. The synergy between DNAzymes and nanoparticles is expected to drive future advancements in these areas.

Furthermore, hybrid systems like DNA@AuNPs provide insights into fundamental concepts like chirality and charge transfer, offering new opportunities to understand DNA catalysis. DNA’s substrate-binding affinity and charge transfer are crucial to its catalytic performance, and these factors are influenced by DNA sequence and conformational flexibility. Increasing active sites or modulating DNA flexibility could optimize binding and catalysis, and altering DNA length, base sequence, or conformational dynamics can enhance efficiency.

Despite groundbreaking discoveries, some aspects of DNA catalysis remain poorly understood, and hybridization introduces additional complexity. Emerging single-molecule techniques, such as single-particle force spectroscopy and single-molecule fluorescence microscopy, offer unprecedented insights at high resolution. Computational approaches, integrated with artificial intelligence and machine learning, may also play a key role in decoding catalytic mechanisms. Future research will focus on the application of DNAzymes in complex biological environments and industrial processes. Improved screening and selection methods will enable the discovery and refinement of these catalytic tools for practical use. DNA’s structural flexibility, sequence programmability, and capacity for hybridization ensure that these catalysts will continue to drive innovation across fields, from diagnostics to therapeutic interventions.

## Data Availability

Not applicable.
